# Biological characters of *Trichogramma dendrolimi* (Hymenoptera: Trichogrammatidae) reared *in vitro* versus *in vivo* for thirty generations

**DOI:** 10.1038/s41598-017-17915-9

**Published:** 2017-12-20

**Authors:** Xin Lü, Shichou Han, Zhigang Li, Liying Li

**Affiliations:** Guangdong Key Laboratory of Animal Conservation and Resource Utilization, Guangdong Public Laboratory of Wild Animal Conservation and Utilization, Guangdong Institute of Applied Biological Resources, Guangzhou, 510260 China

## Abstract

*Trichogramma dendrolimi* which is an economically important biological control agent were reared for 30 generations on a modified artificial medium and natural host. Biological characters were assessed and compared with parasitoids reared *in vivo*. Pupation rate and normal adults rate of *in vitro*–reared parasitoids were significantly higher compared with *in vivo*–reared parasitoids. The adult emergence rate, number of adults produced, and fecundity of *T. dendrolimi* reared *in vitro* were lower than those reared *in vivo*. The percentage of females and longevity did not vary between the two rearing methods. The overall fitness of the parasitoids reared artificially from the first to the 20th generation was higher than of those reared from the 21st generation onwards. No differences were observed in the fitness parameters of parasitoids reared *in vivo* across any of the 30 generations. The results suggest that the modified artificial medium used in this study is suitable for the continuous rearing of *T. dendrolimi* for at least 20 generations, and has the potential for the mass production of these parasitoids for use in biological control. Such a substrate could be examined for use in rearing other parasitoid species that are important in biological control.

## Introduction

*Trichogramma* is a mainstay of augmentative biological control and is annually applied on 15 million hectares across 40 countries worldwide^1^. *Trichogramma dendrolimi* has major economic importance as a biological control agent; this species has a wide host range and is mass produced for biological control programs in China. *In vitro* technologies using artificial host eggs have been developed for the mass rearing of this parasitoid^1^. The original medium developed contains a large proportion (40%) of pupal hemolymph from *Antheraea pernyi* ^2^. To reduce both the content of hemolymph required and the associated costs of its production, an artificial medium that was supplemented with trehalose in sterile water to partially replace the pupal hemolymph was developed by Lü *et al*. and optimized based on an orthogonal array design^3,4^.

Biological traits, such as parasitization capacity, longevity, fecundity, adult size and weight, flight actvity, and searching ability, are generally considered to criteria for assessing the quality of insects reared *in vitro* (reared on artificial media) and *in vivo* (reared on natural hosts)^5,6^. In the case of *Trichogramma*, Cônsoli & Parra showed that females reared on artificial diets have a reduced fecundity compared with those reared on natural hosts^7^, whereas others have reported similar fecundity and longevity between females reared *in vivo* and *in vitro* ^8,9^. Thus, the emergence rate, sex ratio, fecundity, and longevity are used as reproduction parameters for controling the quality of artifically reared *Trichogramma* ^10,11^.

Most studies examining the effects of rearing parasitoids on artificial host eggs versus artificial and/or natural hosts only consider the effects on a single generation^6,9,12,13^, with few examining the effects of continuous culture for several generations^8,14–16^. Meanwhile, even fewer studies have investigated the quality of the insects produced using artificial media by comparing not only biological characters, but also biochemical parameters over multiple generations^6,13^. Lü *et al*. reported the biochemical analyses of *T. dendrolimi* adult carcasses to compare the proportion of nutrients between the artificial medium and a natural host over 10 generations^16^. Therefore, the present study determined the quality of the modified rearing medium by comparing biological parameters, including rates of parasitism, pupation, adult emergence and normal adults, number of adults, percentage of females, fecundity, and longevity, of *T. dendrolimi* reared on artificial medium and those reared on *A. pernyi* eggs for 30 generations.

## Results

### Comparison of *in vitro*- and *in vivo*-reared *T. dendrolimi* over 30 generations

**Figure 1:**
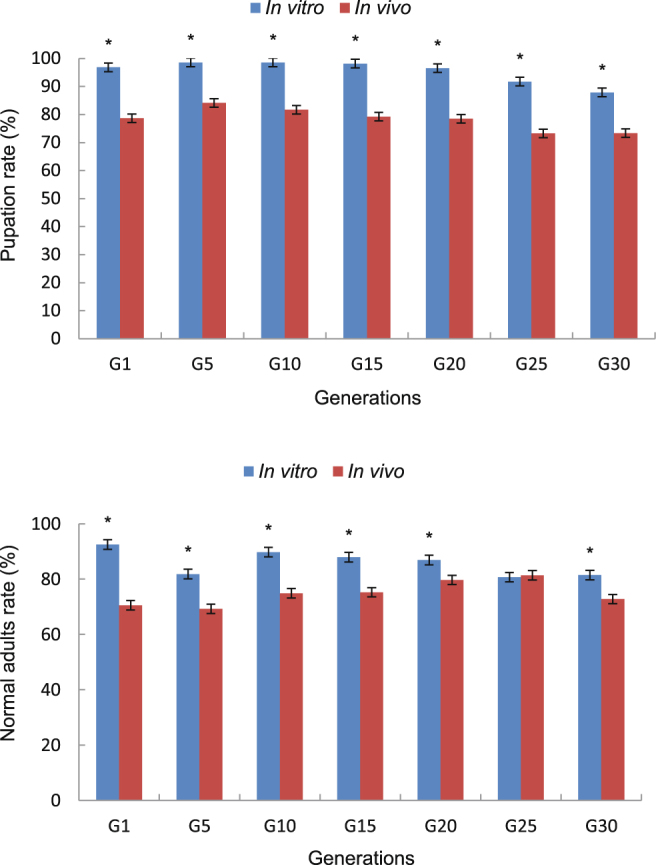
Rates of pupation and normal adults of *Trichogramma dendrolimi* reared *in vitro* and *in vivo* for 30 generations. Means (±SE) were calculated from five replicates. Arcsin transformation (= asin (sqrt (x/100))) where x is a percentage. Data with an asterisk differed significantly according to paired–sample t–tests at *P* = 0.05.

Rates of pupation and normal adults from generation G1 to G30 were significantly higher in the *in vitro* culture compared with *in vivo* culture (Fig. 1), although there was no difference in the rate of normal adults at G25 (Table 1).

**Table 1:** t-values and p-values of paired–sample t–tests for biological parameters comparison of *Trichogramma dendrolimi* readed *in vitro* versus *in vivo*.

Biological parameters	t–value	df	p
Pupation rate (%)	8.102	4	**0.001**
	6.510	4	**0.003**
	6.426	4	**0.003**
	9.223	4	**0.001**
	7.427	4	**0.002**
	5.583	4	**0.005**
	6.268	4	**0.003**
Normal adults rate (%)	6.601	4	**0.003**
	8.309	4	**0.001**
	5.167	4	**0.007**
	4.576	4	**0.010**
	5.426	4	**0.006**
	−0.333	4	0.756
	4.401	4	**0.012**
Emergence rate (%)	−6.452	4	**0.003**
	−14.139	4	**<0.001**
	−5.624	4	**0.005**
	−3.750	4	**0.020**
	−5.733	4	**0.005**
	−27.581	4	**<0.001**
	−14.245	4	**<0.001**
Percentage of females (%)	−10.719	4	**<0.001**
	−3.594	4	**0.023**
	−1.017	4	0.367
	−0.700	4	0.523
	−1.536	4	0.199
	−5.303	4	0.006
	−4.186	4	**0.014**
Number of adults produced	−3.786	4	**0.019**
	−1.329	4	0.255
	−2.850	4	**0.046**
	−4.400	4	**0.012**
	−3.585	4	**0.023**
	−16.452	4	**<0.001**
	−7.475	4	**0.002**
Fecundity	−5.027	29	**0.007**
	−1.006	29	0.371
	−0.5.756	29	**0.005**
	−3.224	29	**0.032**
	−4.008	29	**0.016**
	−8.316	29	**0.001**
	−10.214	29	**0.001**
Longevity (D)	1.798	29	0.083
	−3.474	29	**0.002**
	6.384	29	**<0.001**
	−1.551	29	0.132
	−3.197	29	**0.003**
	−1.795	29	0.083
	−1.780	29	0.086

The bold p-values are considered significantly different.

**Figure 2:**
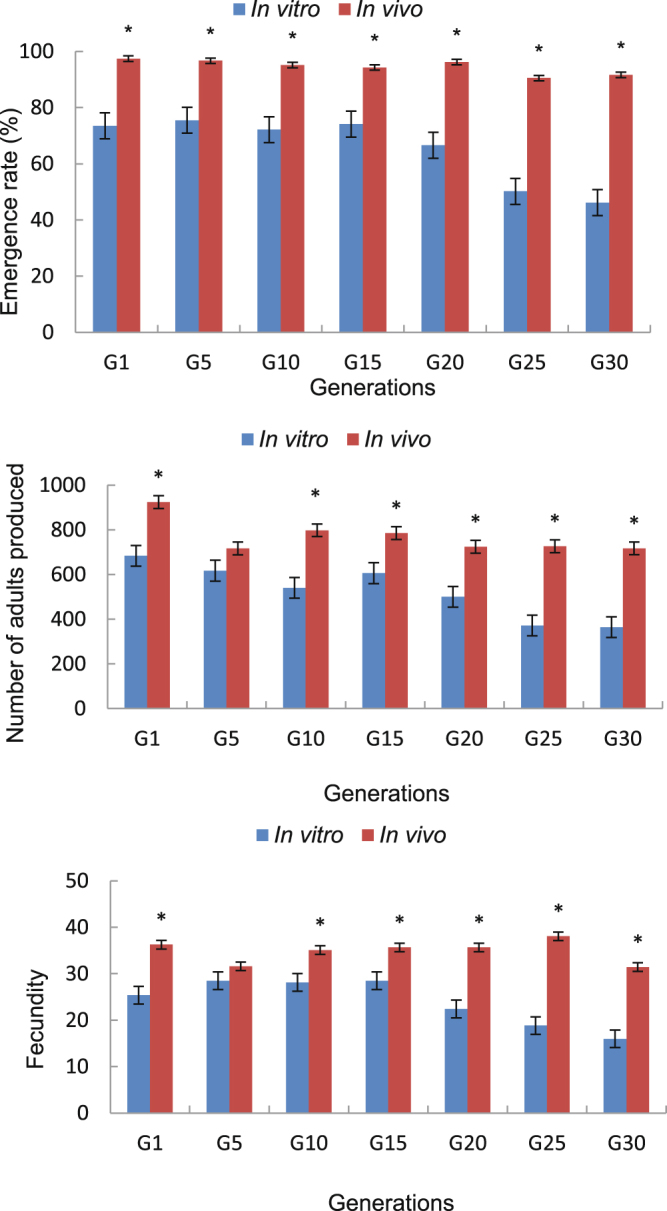
Emergence rate, number of adults produced and fecundity of *Trichogramma dendrolimi* reared *in vitro* and *in vivo* for 30 generations. Means (±SE) were calculated from five replicates. Arcsin transformation (= asin (sqrt (x/100))) where x is a percentage, and Log10 transformation (= log10(x)) where x is data of fecundity and number of adults produced. Data with an asterisk differed significantly according to paired–sample t–tests at *P* = 0.05.

By contrast, the mean rate of adult emergence from pupae, number of adults produced, and fecundity of *T. dendrolimi* reared on artificial medium were lower than for those reared on *A. pernyi* eggs over the 30 generations (Fig. 2) except for G5, where no differences occurred in the number of adults produced and in their fecundity (Table 1).

In the *in vivo* culture, emergence rates were over 90% in all the generations, whereas they were all under 80% in the *in vitro* culture. The number of adults and fecundity of the *in vivo*–reared G30 generation was almost twice that of the *in vitro*–reared insects.

**Figure 3:**
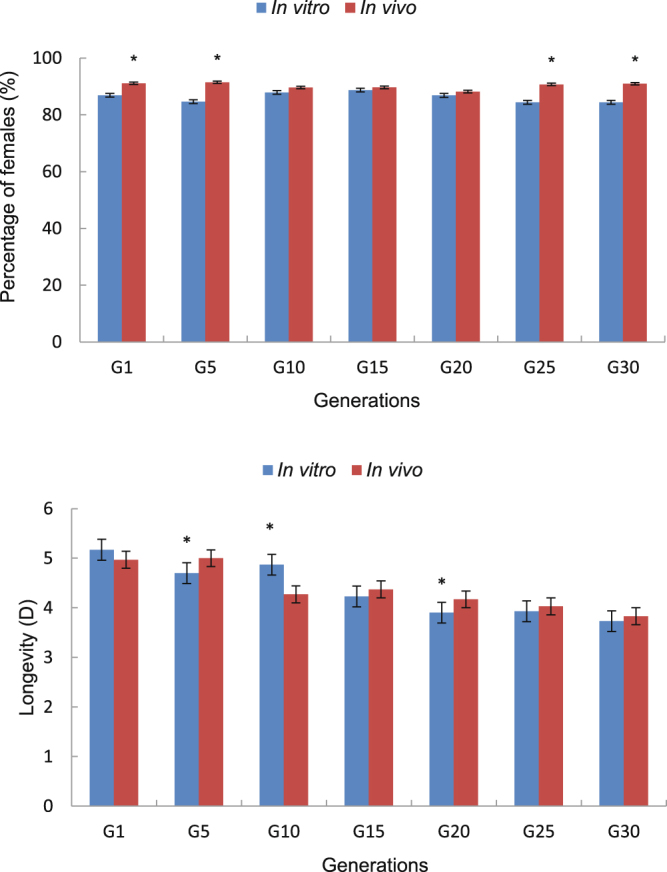
Percentage of females and longevity of *Trichogramma dendrolimi* reared *in vitro* and *in vivo* for 30 generations. Means (±SE) were calculated from five and thirty replicates. Arcsin transformation (= asin (sqrt (x/100))) where x is a percentage, and Log10 transformation (= log10(x)) where x is data of longevity. Data with an asterisk differed significantly according to paired–sample t–tests at *P* = 0.05.

The percentage of females and longevity rates of *T. dendrolimi* adults grown *in vitro* versus *in vivo* were similar across the 30 generations (Fig. 3). The percentage of females in G1, G5, G25, and G30 was significantly greater when reared *in vivo*, and there were significant differences in lifespan of G5, G10 and G20 insects between two rearing methods. However, no differences were found in either lifespan or the percentage of females in *in vivo*– versus *in vitro*–reared insects in the other generations (Table 1).

### Efficacy of *in vitro* versus *in vivo* rearing of *T. dendrolimi* over 30 generations

Parameter values are detailed in Table 2. For *in vitro*–reared insects, there were significant differences in all parameters except for the percentage of females (Table 3). Emergence rate, normal adults rate, number of adults produced, and fecundity of G30 insects decreased by 37.18%, 11.94%, 46.77%, and 37.01%, respectively, compared with G1 parasitoids.

**Table 2:** Biological parameters of *Trichogramma dendrolimi* reared *in vitro* and *in vivo* for 30 generations.

Generations	Biological parameters													
	Pupation rate (%)		Emergence rate (%)		Normal adults rate (%)		Percentage of females (%)		Number of adults produced		Fecundity		Longevity (D)	
	*In vitro*	*In vivo*	*In vitro*	*In vivo*	*In vitro*	*In vivo*	*In vitro*	*In vivo*	*In vitro*	*In vivo*	*In vitro*	*In vivo*	*In vitro*	*In vivo*
G1	96.84 ± 0.81 ab	78.66 ± 1.37 A	73.52 ± 1.65 a	97.41 ± 2.61 A	92.51 ± 0.98 a	70.54 ± 2.89 A	86.91 ± 0.78 a	91.13 ± 1.69 A	683.80 ± 44.68 a	923.4 ± 35.86 A	25.37 ± 1.91 a	36.27 ± 1.62 A	5.17 ± 0.18 a	4.97 ± 0.16 A
G5	98.56 ± 0.73 a	84.15 ± 3.14 A	75.50 ± 0.89 a	96.69 ± 0.80 AB	81.85 ± 1.11 cd	69.26 ± 2.19 A	84.67 ± 1.16 a	91.44 ± 0.73 A	616.60 ± 19.63 ab	715.80 ± 68.46 A	28.49 ± 1.62 a	31.59 ± 2.38 A	4.70 ± 0.20 ab	5.00 ± 0.20 A
G10	98.58 ± 0.60 a	81.70 ± 1.39 A	72.17 ± 1.96 a	95.16 ± 1.47 AB	89.76 ± 1.20 ab	74.86 ± 1.89 AB	87.89 ± 0.97 a	89.64 ± 0.95 A	539.80 ± 41.60 ab	797.80 ± 32.60 A	28.12 ± 1.15 a	35.10 ± 2.53 A	4.87 ± 0.16 ab	4.27 ± 0.17 AB
G15	98.16 ± 0.61 a	79.24 ± 2.44 A	74.15 ± 5.07 a	94.30 ± 1.69 AB	87.92 ± 1.01 b	75.27 ± 3.84 AB	88.74 ± 1.30 a	89.67 ± 0.57 A	605.6 ± 26.90 ab	785.00 ± 32.60 A	28.48 ± 1.42 a	35.66 ± 2.36 A	4.23 ± 0.21 bc	4.37 ± 0.20 AB
G20	96.52 ± 0.55 ab	78.46 ± 3.00 A	66.59 ± 4.42 a	96.27 ± 1.70 AB	86.92 ± 0.25 bc	79.71 ± 1.52 AB	86.85 ± 0.94 a	88.16 ± 0.88 A	499.80 ± 37.48 b	723.80 ± 39.78 A	22.39 ± 2.04 ab	35.67 ± 1.90 A	**3.90 ± 0.19 c**	**4.17 ± 0.19 B**
G25	91.72 ± 0.75 bc	73.26 ± 1.97 A	**50.22 ± 2.92 b**	**90.53 ± 1.16 B**	**80.70 ± 0.84 d**	**81.39 ± 2.14 B**	84.42 ± 1.63 a	90.72 ± 0.52 A	**371.40 ± 23.69 c**	725.60 ± 49.10 A	18.83 ± 1.50 bc	38.07 ± 2.78 A	**3.93 ± 0.19 c**	**4.03 ± 0.18 B**
G30	**87.84 ± 0.71 c**	73.36 ± 3.12 A	**46.19 ± 0.94 b**	91.68 ± 1.72 AB	**81.46 ± 1.55 d**	72.80 ± 1.30 AB	84.40 ± 1.67 a	90.94 ± 0.70 A	**364.00 ± 18.69 c**	716.60 ± 37.72 A	**15.98 ± 0.50 c**	31.43 ± 2.04 A	**3.73 ± 0.14 c**	**3.83 ± 0.14 B**

Data are expressed as the means ± SE based on five replicates (data of fecundity and longevity expressed as the means ± SE based on 30 replicates). Arcsin transformation (= asin (sqrt (x/100))) where x is a percentage; Log10 transformation (= log10(x)) where x is data of fecundity, longevity, and number of adults produced. Means followed by the same letter in the same column were not significantly different at the 5% level (Tukey’s test). The bold values are considered significantly different.

**Table 3:** F-values and p-values of ANOVA for biological parameters of *Trichogramma dendrolimi* readed *in vitro* and *in vivo* for 30 generations.

Biological parameters		F–value	df	p
Pupation rate (%)	*In vitro*	13.074	6, 34	**<0.001**
	*In vivo*	2.488	6, 34	**0.047**
Emergence rate (%)	*In vitro*	15.190	6, 34	**<0.001**
	*In vivo*	2.651	6, 34	**0.037**
Normal adults rate (%)	*In vitro*	18.694	6, 34	**<0.001**
	*In vivo*	3.504	6, 34	**0.010**
Percentage of females (%)	*In vitro*	2.042	6, 34	0.093
	*In vivo*	1.426	6, 34	0.240
Number of adults produced	*In vitro*	16.177	6, 34	**<0.001**
	*In vivo*	2.185	6, 34	0.075
Fecundity	*In vitro*	12.338	6, 209	**<0.001**
	*In vivo*	1.221	6, 209	0.325
Longevity	*In vitro*	8.910	6, 209	**<0.001**
	*In vivo*	5.910	6, 209	**<0.001**

The bold p-values are considered significantly different.

For *in vivo*–reared insects, no differences occurred across generations in any parameter except the rate of emergence and number of normal adults at G25, and females longevity at G20–G30 (Table 3).

## Discussion

To aid the successful and economic rearing of parasitoids for use in biological control programs, there is a need to compare the biological characteristics of *in vitro*–reared insects with those reared in factitious and/or natural hosts, not only over a single generation, but also over continuous generations, especially when focusing on the quality of parasitoids reared on artificial media^8,17^. Therefore, the current study analyzed the biological traits of *T. dendrolimi* successively reared *in vitro* and *in vivo* for 30 generations.

Before *T. dendrolimi* adult emergence began, each egg card was cut open to aid emergence and allow the insects to spread their wings, resulting in fewer deformed adults being obtained on artificial hosts. In fact, the number of normal adults (effective adults) reared *in vitro* were less than *in vivo*. In addition, similar percentage of females and lifespan between the two culture systems demonstrated that it is possible to use the artificial medium tested here in to reproduce *T. dendrolimi* and the efficacy is similar with the natural host eggs.

The lower emergence rates, fewer adults produced and fecundity observed with *in vitro* culture versus *in vivo* culture and previous biochemical results of the adults produced *in vitro*, which showed decreased protein concentrations compared with those reared *in vivo* ^16^, indicated that the medium might not be wholly suitable for mass rearing in terms of its nutrient load compared with natural host eggs. Thus, parasitoids reared on artificial medium appeared to be inferior compared with those reared on *A. pernyi* eggs. The emergence rates of both *T. dendrolimi* and *Trichogramma chilonis* reared *in vitro* were 90% of that reared *in vivo* ^18^, and *in vitro*–reared females of *Trichogramma australicum* produced significantly more progeny than did females reared on natural or factitious hosts for only one generation^9^. *Trichogramma minutum* were reared for 10 generations on an artificial diet that resulted in more deformed females, but also in adults that lived longer, parasitized more *Helicoverpa zea* eggs and had similar emergence rates compared with insects reared *in vivo* on *H. zea* eggs, suggesting, while this medium is nutritionally adequate, additional work is required for it to be suitable for use in mass rearing programs^8^.

Compared with the performances of *T. dendrolimi* reared on the artificial host egg EC–II^19^, the modified medium used herein supported the production of more than 20 generations, with more pupae, normal adults, and females. Dai *et al*. reported the continuous rearing of *T. dendrolimi* on an artificial diet comprising pupal holotissue of *A. pernyi* (30%), egg yolk (14%), skimmed milk (26%), and distilled water (30%) for 41–50 generations, with 60–80% pupation and emergence rates, while obtaining 8% malformed adults^20^. However, Grenier and De Clercq suggested that it is not advisable to maintain entomophagous insects on synthetic diets for many generations, because they may suffer from non–intentional selection, inducing a reduction in genetic variability and deterioration in performance^6^.

Parasitoids of G1–G20 showed fitness to the artificial host compared with G21–G30, with stronger and more numerous normal adults, higher reproductive capacity, - live longer and stable percentage of females. However, almost all the biological parameters decreased after the 20th generation. Nutritional deficiency of the artificial medium should be the major reason of the intergenerational defects. Other than nutrition, inbreeding, which is common in parasitoids, might be one explanation for this decline in fitness^21–23^. Although inbreeding depression in *Trichogramma* appears unlikely^24,25^, the decline in genetic quality could arise because the *in vitro*–reared adults were bred in a small egg card that increased opportunities for inbreeding. Meanwhile, deformed adults that were not removed during the continuous rearing process could have also resulted in the population decline^21^. Therefore, for successful mass production, it would be necessary to rejuvenate the parasitoids every few generations. However, the frequent introduction of new strains for *in vitro* mass production would: (i) require allowing each new strain to adapt to laboratory conditions within a few generations; (ii) carry the risk of misidentification of the introduced strain or species; and (iii) risk introducing pathogens or hyperparasitoids to the breeding system^6^.

In conclusion, based on the biological parameters examined here and combined with previous biochemical analyses^16^, our studies indicate that the modified artificial medium was suitable for the development of *T. dendrolimi* from eggs to adults, and supported adult survival and continuous reproduction for at least 20 generations. Liu *et al*. reported there is no significant difference in parasitism and development of *T. dendrolimi* reared in the lyophilized diet that either stored under −16 °C for 522 days or stored at room temperature for 140 days in comparison with that developed in fresh diet^26^. It has been estimated that the costs for producing *Trichogramma* on artificial media are 50% lower than on their factitious or natural hosts^20^. The artificial medium could be used to overcome shortages in the supply of lepidopteran host eggs and reduce parasitoid production costs. It also has the potential for use as an artificial host for the large–scale production of this parasitoid. However, there is a need to test the efficacy of the parasitoids reared using this medium against target pests in the field. Future studies should also examine the continuous rearing of other species of natural enemies, and the impact on their quality, using the methods described herein.

## Materials and Methods

### Stock culture

*Trichogramma dendrolimi* was originally collected from the Institute of Plant and Environment Protection, Beijing Academy of Agricultural and Forestry Sciences, Beijing, China, the same as Lü *et al*.^3,4,16^ reported previously. In the laboratory, *T. dendrolimi* stock cultures were reared on eggs of *A. pernyi* as a factitious host. Climatic conditions were 27 ± 1 °C, 75% ± 5% relative humidity (RH) and a 16:8 L:D photoperiod.

### Artificial medium preparation

The artificial medium used in this study was the modified artificial medium developed by Lü *et al*.^4^ (Table 4). The artificial medium was prepared as described previously^3^. Pupal hemolymph was obtained from live *A. pernyi* pupae that were immersed in a water bath at 60 °C for 10 min to avoid melanization of the hemolymph. After sterilization of the pupae surface with 75% ethanol, the hemolymph was collected by pressing the pupae under sterile conditions. Neisenheimer’s salt solution was prepared with NaCl 7.5 g, KCl 0.1 g, CaCl_2_ 0.2 g, NaHCO_3_ 0.2 g, and 1000 mL of distilled water and used after sterilization.

**Table 4:** Composition of the artificial medium used for the *in vitro* rearing of *Trichogramma dendrolimi*.

Component	Content
*A. pernyi* pupal hemolymph	3.0 ml
Egg yolk	2.5 ml
10% malted milk solution	1 ml
Neisenheimer’s salt solution	1 ml
Trehalose	0.1 g
Sterile water	1.5 ml

### Preparation of artificial egg cards and *A. pernyi* egg cards

For artificial egg cards, the preparation referred to the work by Lü *et al*.^16^. Twenty semispherical domes (2 mm–3 mm in diameter × 3 mm high) were produced by pressing a heated glass rod onto one half of a sheet (8 cm × 7 cm) of polyethylene and polypropylene copolymer film (30 µm thick) through a plastic semispherical mold. After the sheets were sterilized by UV irradiation, 4 µL medium was transferred to each dome using a pipette. The half of the sheet containing the domes (convex side) to be exposed to oviposition by the parasitoid was folded over the other half and sealed using a plastic sealer, so that the concave side and the bottom piece of film provided sufficient space to allow aeration for parasitoid development. Afterward, the external surface of the egg cards was treated with 10% (wt/vol) polyvinyl alcohol to stimulate oviposition. For *A. pernyi* egg cards, twenty eggs were attached to cardboard strips (2 cm × 2 cm) with 10% (wt/vol) polyvinyl alcohol.

### *In vitro* and *in vivo* rearing

For the first generation, twenty artificial egg cards and twenty *A. pernyi* egg cards were placed in a plastic tray (30 cm × 20 cm × 5 cm) for exposure to *T. dendrolimi* adults of the same generation for 24 h. Parasitoids of both sexes were released in the trays using a ratio of parasitoids to artificial eggs of 6:1. Sex ratios were approximately 8:1 in all five replicates (one tray corresponded to one replicate). Trays were placed in climatic incubators set at 27 ± 1 °C, 75% ± 5% RH and a 16:8 L:D photoperiod. After 24 h of exposure, the egg cards were taken out and the wasps were removed. From the third day after parasitization, the egg cards were monitored for parasitoid development under a binocular microscope. Before *T. dendrolimi* adult emergence began, each egg card was inserted into a glass tube (3 cm in diameter × 9 cm high), which was covered by a cotton cloth with a rubber band. Each artificial egg card was cut open to aid emergence before adults emergence. When adults emerged, a new egg card was inserted into the glass tube for rearing of the next generation. Adults of two egg cards from each tray were used for biological assessment.

### Biological parameters assessed

During the *in vitro* and *in vivo* rearing process, the number of larvae, pupae, total adults, and male versus female adults were counted; the fecundity of female adults produced from both cultures was recorded every five generations.

Rates of pupation and adult emergence, the proportion of normal adults (i.e., adults with normal wings and abdomen) referred to Lü *et al*.^3,4^ and percentage of females were calculated as follows:

$$\begin{array}{rcl}{\rm{Pupation}}\,{\rm{rate}} & = & ({\rm{number}}\,{\rm{of}}\,\text{pupae}/\text{total}\,{\rm{number}}\,{\rm{of}}\,{\rm{larvae}}\,{\rm{observed}}\,{\rm{per}}\,{\rm{egg}}\,{\rm{card}})\times 100.\\ {\rm{Emergence}}\,{\rm{rate}} & = & ({\rm{number}}\,{\rm{of}}\,\text{adults}/\text{total}\,{\rm{number}}\,{\rm{of}}\,{\rm{pupae}}\,{\rm{observed}}\,{\rm{per}}\,{\rm{egg}}\,{\rm{card}})\times 100.\\ {\rm{Normal}}\,{\rm{adults}}\,{\rm{rate}} & = & ({\rm{number}}\,{\rm{of}}\,{\rm{normal}}\,\text{adults}/\text{total}\,{\rm{number}}\,{\rm{of}}\,{\rm{adults}}\,{\rm{observed}}\,{\rm{per}}\,{\rm{egg}}\,{\rm{card}})\times 100.\\ {\rm{Number}}\,{\rm{of}}\,{\rm{adults}}\,{\rm{produced}} & = & {\rm{total}}\,{\rm{number}}\,{\rm{of}}\,{\rm{adults}}\,{\rm{observed}}\,{\rm{to}}\,{\rm{emerge}}\,{\rm{from}}\,{\rm{five}}\,{\rm{replicates}}\,({\rm{eggcards}})/5.\\ {\rm{Percentage}}\,{\rm{of}}\,{\rm{females}} & = & {\rm{total}}\,{\rm{number}}\,{\rm{of}}\,{\rm{female}}\,{\rm{adults}}\,\text{observed}/\text{total}\,{\rm{number}}\,{\rm{of}}\,{\rm{adults}}\,{\rm{observed}}\times 100.\end{array}$$

Fecundity was measured by holding newly emerged females over a glass tube (3 cm in diameter × 9 cm high) containing an artificial egg card or *A. pernyi* egg card with 35 eggs. The number of larvae and pupae were counted until the death of the female and the mean fecundity of each female was then calculated. The date of emergence and death of the females were also recorded to calculate longevity. Thirty *in vitro*– and thirty *in vivo*–reared females were tested every 5 generations.

### Statistical analysis

Mean pupation, emergence, normal adults and female adults percentage, mean number of adults produced, fecundity and longevity for this parasitoid reared in i*n vitro* and *in vivo* were compared using one-way analysis of variance (ANOVA) and Tukey’s test across continuous rearing *T. dendrolimi* for 30 generations. Paired–sample t–tests were used to analyze differences between the artificial medium (*in vitro*) and *A. pernyi* eggs (*in vivo*) groups. Before analysis, percentage data were arcsine square root–transformed, data of fecundity, longevity, and number of adults produced were log10-transformed to fit a normally distributed. In all experiments, differences among means were considered significant at P < 0.05. Statistical analyses were conducted by using SPSS 17.0 software (SPSS Inc. Chicago, IL, USA).

### Data availability

All data generated or analysed during this study are included in this published article.

## References

[1] Vinson, S. B., Greenberg, S. M., Liu, T. X., Rao, A. & Volosciuc, L. F. *Biological control of pests using Trichogramma: current status and perspectives*. (Northwest A & F University Press, Yangling, Shanxi, China, 2015).

[2] Li LY (1986). *In vitro* rearing of *Trichogramma* spp. and *Anastatus* sp. in artificial “egg” and the methods of mass production. Les Colloques de l’INRA..

[3] Lü X, Han SC, Li LY, Grenier S, De Clercq P (2013). The potential of trehalose to replace insect hemolymph in artificial media for *Trichogramma dendrolimi* Matsumura (Hymenoptera: Trichogrammatidae). Insect Sci..

[4] Lü X, Han SC, De Clercq P, Dai JQ, Li LY (2014). Orthogonal array design for optimization of an artificial medium for *in vitro* rearing of *Trichogramma dendrolimi*. Entomol. Exp. Appl..

[5] Kazmer DJ, Luck RF (1995). Field tests of the size–fitness hypothesis in the egg parasitoid *Trichogramma pretiosum*. Ecology.

[6] Grenier, S. & De Clercq, P. In *Quality control and production of biological control agents: Theory and* Testing *Procedures* (eds van Lenteren, J. C.), 115–131 (CABI Publishing, Wallingford, 2003).

[7] Cônsoli FL, Parra JRP (1996). Biology of *Trichogramma galloi* Zucchi and *T. pretiosum* Riley reared “*in vivo*” and “*in vitro*”. Ann. Entomol. Soc. Am..

[8] Nordlund DA, Wu ZX, Greenberg SM (1997). *In vitro* rearing of *Trichogramma minutum* Riley (Hymenoptera: Trichogrammatidae) for ten generations, with quality assessment comparisons of *in vitro* and *in vivo* reared adults. Biol. Control.

[9] Nurindah GG, Cribb BW (1997). Oviposition behaviour and reproductive performance of *Trichogramma australicum* Girault (Hymenoptera: Trichogrammatidae) reared in artificial diet. Aust. J. Entomol..

[10] Cerutti F, Bigler F (1995). Quality assessment of *Trichogramma brassicae* in the laboratory. Entomol. Exp. Appl..

[11] Dutton A, Cerutti F, Bigler F (1996). Quality and environmental factors affecting *Trichogramma brassicae* (Hym: Trichogrammatidae) in the laboratory and field conditions. Entomophaga.

[12] Grenier S, Yang H, Guillaud J, Chapelle L (1995). Comparative development and biochemical analyses of *Trichogramma* (Trichogrammatidae: Hymenoptera) in artificial media with hemolymph or devoid on insect components. Comparat. Biochem. Physiol..

[13] Cônsoli, F. L. & Grenier, S. In *Egg Parasitoids in Agroecosystems with Emphasis on* Trichogramma (eds Cônsoli, F. L., Parra, J. R. P. & Zucchi, R. A.), 293–313 (Springer, Dordrecht, The Netherlands, 2010).

[14] De Clercq P, Degheele D (1992). A meat–based diet for rearing the predatory stinkbugs *Podisus maculiventris* and *Podisus sagittal* (Het.: Pentatomidae). Entomophaga.

[15] De Clercq P, Degheele D (1993). Quality assessment of the predatory bugs *Podisus maculiventris* (Say) and *Podisus sagittal* (Fab.) (Heteroptera: Pentatomidae) after prolonged rearing on a meat–based artificial diet. Biocontrol Sci. Techn..

[16] Lü X, Han S, Li LY (2015). Biochemical analyses of *Trichogramma dendrolimi* (Hymenoptera: Trichogrammatidae) *in vitro* and *in vivo* rearing for 10 generations. Fla. Entomol..

[17] Gao YG, Dai KJ, Shong LS (1982). *Trichogramma* sp. and their utilization in. People’s Republic of China. Les Colloques de l’INRA.

[18] Feng JG, Tao X, Zhang AS, Yu Y, Zhang CW (1999). Study on using *Trichogramma* spp. on artificial host egg to control corn pests. Chin. J. Biol. Control.

[19] Ma, Z. J., Xu, K. J. & Chen, B. Q. In *Studies of artificial host egg for* Trichogramma. (eds Hubei Province Cooperative Research Group of Artificial Host Eggs for *Trichogramma*), 229–236 (Wuhan University Press, Wuhan, China, 1987).

[20] Dai KJ (1991). Research on technology of industrial production of the artificial host egg of *Trichogramma*. Les Colloques de l’INRA.

[21] Suzuki Y, Hiehata K (1985). Mating systems and sex ratio in the egg parasitoids, *Trichogramma dendrolini* and *T. papilionis* (Hymenoptera: Trichogrammatidae). Anim. Behav..

[22] Kazmer DJ, Luck RE (1991). The genetic–mating structure of natural and agricultural populations of *Trichogramma*. Les Colloques de I’INRA.

[23] Hardy ICW (1994). Sex ratio and mating structure in the parasitoid Hymenoptera. Oikos.

[24] Li LY, Zhang YH (1980). The study of inbreeding in *Trichogramma*. Nat. Enemies Insects.

[25] Sorati M, Newman M, Hoffmann AA (1996). Inbreeding and incompatibility in *Trichogramma* nr. *brassicae*: evidence and implications for quality control. Entomol. Exp. Appl..

[26] Liu WH, Chen QX, Han SC (1993). Lyophilized diet for *in vitro* rearing *Trichogramma dendrolimi*. Nat. Enemies Insects.

